# Procalcitonin, presepsin, pro-adrenomedullin, fibrin degradation products, and lactate in early diagnosis and prognosis of septic patients newly admitted to the intermediate care unit from the emergency department

**DOI:** 10.1186/cc12917

**Published:** 2013-11-05

**Authors:** Filippo Mearelli, Nicola Fiotti, Nicola Altamura, Irene Paoli, Chiara Casarsa, Maurizio Ruscio, Gianni Biolo

**Affiliations:** 1 Unit of Clinica Medica Generale e Terapia Medica, Department of Medical Surgical and Health Sciences, University of Trieste, Italy; 2 Laboratory Medicine Ospedale Sant' Antonio, San Daniele Del Friuli, Italy

## Background

More than 50% of all septic patients admitted to intensive care departments derive from intermediate care units (INCU). Biomarkers represent the most promising tool for early diagnosis of sepsis; but their accuracy in INCU has been largely disregarded [1]. Moreover, given the complexity of the septic pathophysiology, a panel of biomarkers could be more effective than a single one. For this reason we tested acute phase protein, cell surface, vasotonous related, coagulation system, and tissue hypoxia markers in early ruling in/out of sepsis in patients suffering from systemic inflammatory response syndrome (SIRS) [2-5].

## Materials and methods

This prospective observational study included all SIRS [5] patients newly admitted to a medical ward from February to May 2012. Cases were diagnosed as sepsis or non-infective SIRS by clinical examination, cultures of the biological fluid, and imaging during a 7-day follow-up. Investigators were blinded to biomarker results. Survivors at 7 and 30 days were also assessed. Samples for procalcitonin (PCT), presepsin (sCD14-ST), pro-adrenomedullin (PRO-ADM), fibrin degradation products (FDP) and lactate were collected within 4 hours of admission. Their role in predicting diagnosis and survival, alone or in combination, have been investigated by receiver operating characteristic (ROC) curve, Youden index, relative risk and binary logistic regression.

## Results

**Figure 1:**
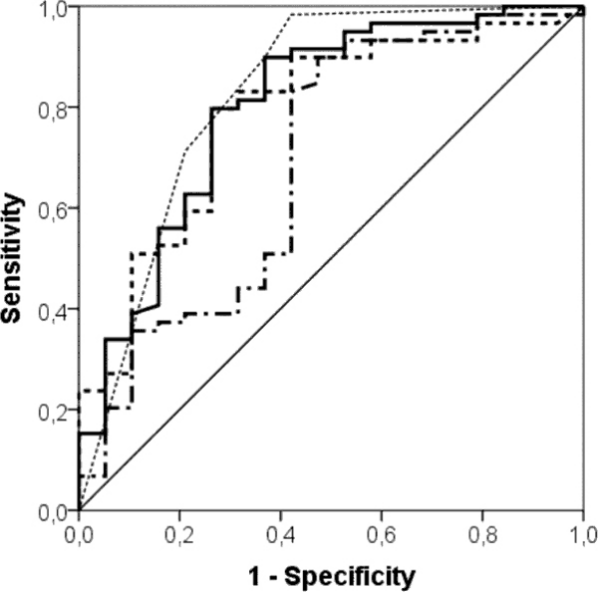
**ROC curves of PCT, FDP, sCD-14ST, and the combination of FDP + PCT**. Solid thick line, PCT; dashed line, FDP; dotted line, PCT+FDP; dot-dash line, sCD-14ST; thin solid line, reference line.

**Figure 2:**
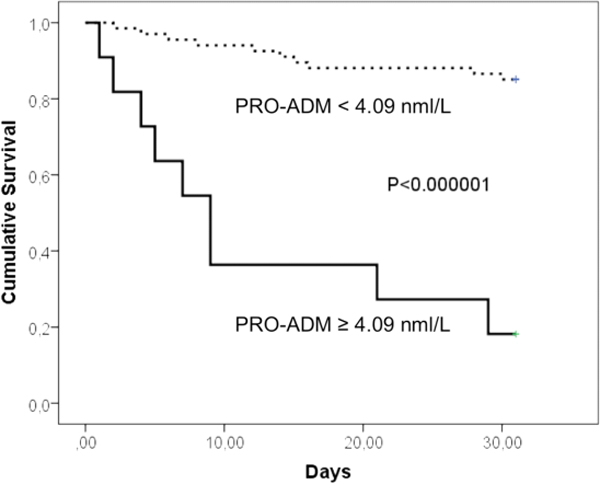
**Thirty-day survival curve (Kaplan-Meier) according to PRO-ADM levels**.

Among the 60 sepsis patients (microbiological and clinical sepsis), the most common sites of infection were the lung (67%), urinary tract (17%), abdomen (5%), and skin (8%). The sepsis group had significantly higher levels of PCT, sCD14-ST and FDP than the non-infective SIRS group. The area under the ROC was 0.80, 0.78, and 0.67 for FDP, PCT, and sCD14-ST respectively. Main results are reported in Table 1: the combination of FDP and PCT detected correctly 10 more cases, leaving misdiagnosed only nine out of 80 patients. ROC curves are reported in Figure 1. sCD14-ST (cutoff 1.317 ng/ml, OR 12.2 (95% CI 2.6 to 55.5) *P *= 0.0002) and lactate (cutoff 20 mg/dl OR 11.9 (95% CI 2.23 to 62.5) *P *= 0.001) were comparable in predicting 7-day survival. Mortality at 30 days was significantly higher in patients with PRO-ADM level ≥4.09 nmol/l (OR 26 (95% CI 4.8 to 143) *P *= 0.000002). The Kaplan-Meier curves for PRO-ADM are reported in Figure 2.

**Table 1:** 

Biomarker	Cutoff	Sensitivity	Specificity	PPV	NPV	PLR	NLR	Accuracy
PRO-ADM	0.2 nmol/l	83	37	80	41	1.3	0.5	72
PCT	0.1 ng/ml	80	74	90	54	3.0	0.2	78
sCD14-ST	0.407 ng/ml	90	50	84	62	1.8	0.2	80
FDP	180 ng/ml	80	70	89	53	2.6	0.2	77
FDP + PCT	180 + 0.1 ng/ml	95	68	90	81	3	0.075	88

## Conclusions

In intermediate care setting patients, the combination of FDP and PCT could be useful for an early discrimination of sepsis from non-infective SIRS. PRO-ADM, sCD14-ST, and lactate should be considered as early indicators of more intensive ward care and precocious ICU admission.
